# Accounting for Fairness in a Two-Stage Stochastic Programming Model for Kidney Exchange Programs

**DOI:** 10.3390/ijerph15071491

**Published:** 2018-07-14

**Authors:** Hyunwoo Lee, Seokhyun Chung, Taesu Cheong, Sang Hwa Song

**Affiliations:** 1 School of Industrial Management Engineering, Korea University, Seoul 02841, Korea; lhw0429@korea.ac.kr (H.L.); csh98016@gmail.com (S.C.); 2 Graduate School of Logistics, Incheon National University, Incheon 22012, Korea

**Keywords:** kidney exchange program, unfairness indicator, exceptional event, two-stage stochastic programming

## Abstract

Kidney exchange programs, which allow a potential living donor whose kidney is incompatible with his or her intended recipient to donate a kidney to another patient in return for a kidney that is compatible for their intended recipient, usually aims to maximize the number of possible kidney exchanges or the total utility of the program. However, the fairness of these exchanges is an issue that has often been ignored. In this paper, as a way to overcome the problems arising in previous studies, we take fairness to be the degree to which individual patient-donor pairs feel satisfied, rather than the extent to which the exchange increases social benefits. A kidney exchange has to occur on the basis of the value of the kidneys themselves because the process is similar to bartering. If the matched kidneys are not of the level expected by the patient-donor pairs involved, the match may break and the kidney exchange transplantation may fail. This study attempts to classify possible scenarios for such failures and incorporate these into a stochastic programming framework. We apply a two-stage stochastic programming method using total utility in the first stage and the sum of the penalties for failure in the second stage when an exceptional event occurs. Computational results are provided to demonstrate the improvement of the proposed model compared to that of previous deterministic models.

## 1. Introduction

Kidney exchange programs (KEPs) offer a new opportunity for patients on a kidney transplantation list to find suitable kidneys. Because everyone is born with two kidneys and can survive with only one, kidney transplantations are possible. There is a higher chance of a match in family members, so kidney transplantations often occur among family members. However, problems arise when family members have kidneys that are incompatible with the patient. In this case, a kidney exchange program is a possible solution. Kidney exchanges can occur when there are two incompatible patient-donor pairs, but the donors are compatible with the patient in the other pairing. Kidney exchanges were introduced in 1986 by Felix Rappaport [1] and first performed in South Korea in 1991 [2,3,4]. Many countries, including Switzerland [5], the UK [6], and the USA [7,8,9], have since performed kidney exchange transplantations. We here remark that the Korea Centers for Disease Control & Prevention (KCDC) has recently reviewed and discussed a plan for the development of an integrated IT healthcare platform for the KEP, and hospitals can participate in the provision of the essential information for their renal disease sufferers with their medical records to the platform. Thus, the KCDC believes that the nationwide KEP platform would possibly provide good matching plans to maximize social utility.

Operations research (OR) technique has been applied extensively across healthcare issues [10], and KEP is one of the important issues where OR technique has been used. KEP is typically modeled as an integer linear programing network optimization problem. Depending on the nature of KEP, a lot of deterministic models for the optimization of these programs have been designed. However, considering that the risk of uncertainty in KEP has not been discussed much, it is still an ongoing issue. The risk of uncertainty in KEP can lead to failure in matching, which means that a surgery cannot be done. In this study, we use the Stochastic Programming (SP) [11,12] framework to consider failure scenarios before matching takes place. In particular, a two-stage stochastic programming is a promising method for this purpose, in that it incorporates the effect of data uncertainty in the second stage. We list possible exceptional events and classify them as two types of failure scenarios, i.e., node failure and arc failure. The former occurs within a single pair and the latter occurs between pairs. Node failure can occur when the condition of the patient in one of the pairs seriously deteriorates. Arc failure can occur when one of two pairs changes its mind, which means when a pair feels unsatisfied with its matching. Because a kidney exchange is a form of bartering, the patient-donor pairs play the role of customers. Depending on the nature of the barter, a single patient-donor pair may refuse to go through with a particular transplantation. We consider the fairness issue at this point and define fairness in different ways. We propose the notion of personal fairness, something which previous studies have not considered much. Personal fairness is the extent to which individual patient-donor pairs feel satisfied with the exchange. We propose a value indicator, unfairness, to represent the level of satisfaction. We give the penalty value to two representative examples of node failure and arc failure to reflect their effect in the second stage of the SP framework.

**Figure 1:**
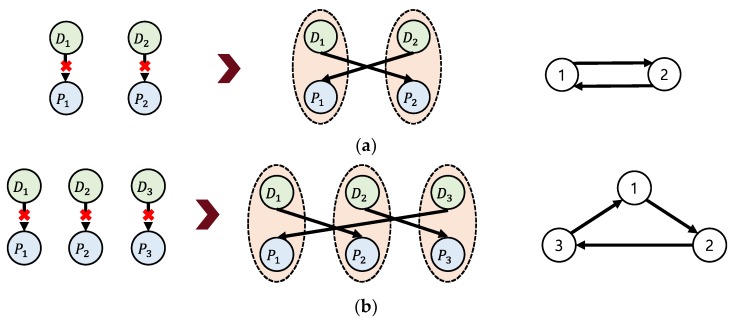
Illustration of (**a**) two-pair and (**b**) three-pair kidney exchanges.

KEP is typically designed as follows. Figure 1 illustrates how incompatible patient-donor pairs can be organized to allow successful surgery to take place by exchanging transplants. Figure 1a illustrates the process by which two incompatible pairs exchange kidneys. If the donor of Pair 1 and the patient of Pair 2 are compatible and the donor of Pair 2 and the patient of Pair 1 are compatible, surgery can take place. Likewise, three incompatible patient-donor pairs can also be matched by exchanging kidneys (Figure 1b).

**Figure 2:**
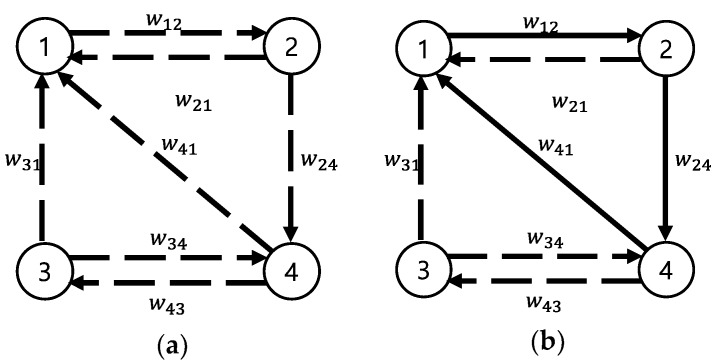
Example of (**a**) a kidney exchange program (KEP) graph and (**b**) three-pair kidney exchanges.

The general KEP model can be expressed by directed graph (V,A) referred to as a *KEP graph*—with set of vertices V and set of arcs A (Figure 2). Vertex i∈V represents an incompatible patient-donor pair and arc (i,j)∈A represents compatibility between pairs (i,j). $w_{ij}$ represents the weight of arc (i,j)∈A, indicating the likelihood of surgical success. Figure 2a presents possible links as dotted lines, while Figure 2b presents a constructed cycle with vertices {1,2,4}⊂V and arcs {(1,2),(2,4),(1,4)}⊂A. A deterministic model for a KEP finds the set of cycles with the maximum weight using objective functions to maximize the sum of the weights.

The following are some of the studies that considered deterministic mathematical models. Roth et al. [13] proposed both edge and cycle formulations for KEPs. Abraham et al. [14] proposed a clearing algorithm that solves established models. Constantino et al. [15] suggested two compact formulations, an edge-assignment (EA) formulation and an extended edge (EE) formulation. Yuh et al. [16] applied the reformulation-linearization technique (RLT) to the EA and EE formulations for deriving a tighter and more compact formulation. The models in these previous studies use objective functions to increase the utility of the program as a whole. However, these models do not reflect equity as experienced by the individuals involved, nor did they attempt to incorporate the risk of failure.

Past research has identified several different perspectives on fairness in terms of KEPs. For example, Bertsimas et al. [17] raised the issue of defining fairness in relation to kidney exchange transplants. They suggested that the concept of fairness can vary depending on the focused target, whether it be the individual or society as a whole. Indeed, most previous studies have defined fair exchanges as being those that increase the overall social benefit by prioritizing difficult-to-match pairs [18]. This method achieves high levels of social fairness but fails to reflect the characteristics of bartering, in which individuals feel the exchange is fair when they are satisfied with it.

The risk of not considering personal fairness is that it can directly lead to the failure of surgery. A single patient-donor pair may refuse to go through with a particular transplantation when they feel unfair, even though the matched kidney is compatible, in order to wait for a better one. In fact, many kidneys that are medically compatible with some of the patients on transplant waiting lists are left unselected and unused [19,20]. Sabouri et al. [21] suggested screening strategies for identifying patients who are ineligible for transplants. These studies imply that there are kidneys which are not suitable for transplant. The attractiveness of the exchange depends on the condition and the value of the kidney; based on these criteria, the patient-donor pair make a choice without considering the medical situation. Therefore, by considering the satisfaction of the individuals involved, it is possible to minimize the amount of unused kidneys and to reduce matching failure. 

It is important to minimize matching failure in a KEP because a single failure can have a flow-on affect that disrupts other possible transplants as well. Previous studies have focused on failure in the context of the kidney exchange problem. For example, Zheng et al. [22] were the first to consider arc failure, while Dickerson et al. [23] mathematically outlined the impact of failure on exchange cycles and chains. Alvelos et al. [24] proposed a compact integer programming model considering failure probabilities and they assumed that all arc and vertices have the same failure probabilities, while our study specified and considered individual pair’s characteristics and the effect of failure with the penalty value. Our work has uniqueness and advantage in its specified approach.

There have been some studies applying a stochastic and heuristic approach. Awasthi et al. [25] proposed online stochastic optimization with trajectory-based algorithms. Dubey et al. [26] utilized ant-lion optimization algorithm as a meta-heuristic approach to solve KEP.

Stochastic programming which is mentioned earlier can be a key to the limitation of uncertainty issues by utilizing different objective functions at each stage. A more robust model can be developed through using an objective function that increases total utility in the first stage, and an objective function that decreases the penalty for failure in the second stage. Because of the presence of the two objective functions, we expect two measurable improvements in this model. One is robustness to exceptional events, and the other is individual satisfaction while maintaining social utility.

In this paper, we propose a two-stage SP model that considers exceptional events that have not been included in previous deterministic models. We develop this two-stage SP model based on the compact EA model introduced by Constantino et al. [15]. We redefine fairness as personal fairness in terms of an individual pair’s satisfaction and establish an indicator of unfairness. We classify failure scenarios and incorporate them into the second stage to obtain a more robust model. Computational results are provided to demonstrate the improvement of this model compared to the previous deterministic model. The contributions of our study can be summarized as follows.

We propose a stage-based SP model that reflects exceptional events before matching takes place. We consider failure scenarios in the second stage and classify failure scenarios as either arc failure or node failure. Experimental results illustrate that this stochastic model is more robust to failure than deterministic models when an exceptional situation occurs. This model thus proves that failure can be taken into account before matching, and this technique can be extended in many ways by adding failure scenarios.

We redefine and formalize fairness within a kidney exchange program as personal fairness, which is a measure of how satisfied individual pairs feel during an exchange. This is a unique approach compared to previous studies, which have defined fairness in terms of increasing social welfare. As such, this study proposes a new model that considers the nature of bartering. We propose an unfairness indicator and set a penalty function with this indicator as an input variable. The SP model that considers personal fairness produces lower total unfairness levels than that of existing deterministic models.

The rest of this paper is organized as follows. Section 2 briefly introduces an overview of stochastic programming. Section 3 then summarizes the two-stage stochastic programming approach to KEPs, illustrates how failure scenarios are classified and incorporated, and presents the stochastic formulation. Following this, Section 4 presents computation results to assess the performance of our model. Finally, we conclude the study in Section 5.

## 2. Stochastic Programming Overview 

Stochastic programming is a framework used to model optimization problems that involve uncertainty. Unlike deterministic optimization problems, which are formulated with known parameters, stochastic programming incorporates unknown parameters. The general SP model can be presented as

(1) $$\begin{array}{cc} \begin{array}{c} \mathrm{Maximize} \end{array} & c^{T}x+E_{\xi }\left[Q\left(x,\xi \right)\right]\\ \mathrm{Subject}\;\mathrm{to} & Ax=b,\\ & x\;\geq 0,\\ & Q\left(x,\xi \right)=\;max\;\{q^{T}y\;|\;Wy=h-Tx,y\;\geq 0\}. \end{array}$$

Model (1) consists of two sections: first-stage and second-stage decisions. Here, x is the first-stage decision variable, and y is the second-stage variable. When the first-stage decision is made, after which a random event occurs affecting the outcome of the first-stage decision, a recourse decision can then be made in the second stage that compensates for any negative effects. $q^{T}y$ is the cost of recourse action and is added up in the objective function. This second term in the objective function tries to repair the decisions which is made before the consideration of a random event. Through this procedure, SP can incorporate the first-stage decision’s effect as well as the second-stage decision’s effect. SP has a set of random data and random vector ξ is used to represent random events with a certain probability. ξ is the vector formed by components $q^{T},\;h^{T},$ and T, and $E_{\xi }$ denotes mathematical expectations with respect to ξ.

**Figure 3:**
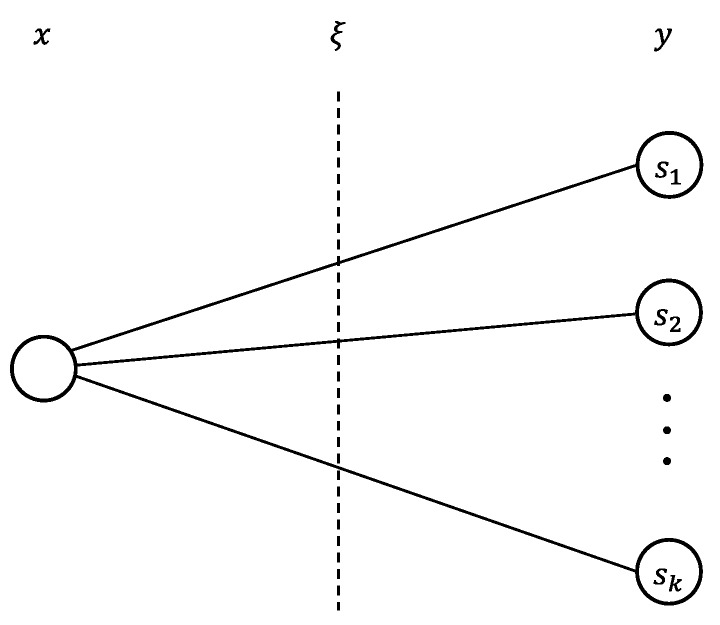
Scenario representation.

Figure 3 illustrates how scenarios are generated. In two-stage stochastic programming, random vector ξ can be represented with respect to scenario k∈K. Each scenario k has the probability of occurrence $p_{k}$, and the sum of $p_{k}$ with respect to k∈K is 1. The second decision variable $y_{k}$ appears in the objective function, and $q_{k},\;T_{k},$ and $h_{k}$, which are parameters for scenario k, appear in the mathematical formulation.

However, Q(x,ξ) in Model (1) can lead to a nonlinear model if Q(x,ξ) is continuous over ξ. Therefore, the model should be discretized for ξ as follows.

(2) $$\begin{array}{cc} \mathrm{Maximize} & c^{T}x+\underset{k\in K}{\sum }p_{k}q_{k}^{T}y_{k}\\ \mathrm{Subjiect}\;\mathrm{to} & Ax=b,\\ & T_{k}x+\;Wy_{k}=h_{k},\;\forall k\in K\\ & x\geq 0,\;y_{k}\geq 0,\;\forall k\in K \end{array}\;$$

where k indicates the index of the scenarios, and $p_{k},\;q_{k},$ and $y_{k}$ are the probability of scenario k, the parameter vector related to scenario k, and the second stage variable of scenario k, respectively. Model (2) is a compact formulation of a two-stage stochastic programming model. In this study, the scenarios are possible cases of failure in kidney exchanges. At the first stage, we consider an objective function that maximizes the sum of weights. At the second stage, we consider causes of failure and set the probability of each scenario k. We establish penalty value $q_{k}$ with respect to $y_{k}$ to incorporate the first stage’s objective function and the second stage’s failure scenarios. We derive a two-stage stochastic programming model in Section 3 based on the well-known EA model by adding failure scenario information; we also demonstrate how the scenarios are classified and generated.

## 3. Two-Stage Stochastic Programming for Kidney Exchange Programs

### 3.1. Scenario Generation

**Figure 4:**
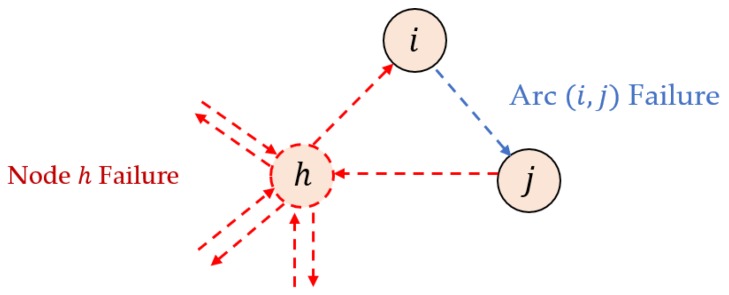
Illustration of the two types of failure in the KEP graph.

In this section, we explain how we generate and classify failure scenarios. The KEP graph consists of two elements, nodes (vertices) and arcs (edges). Nodes represent an incompatible patient-donor pair and arcs represent compatibility between two pairs. Failure may occur between two pairs and within a pair. Therefore, failure scenarios for exceptional events are largely classified into two types of failure: node failure and arc failure. Node failure occurs if there is a problem in the patient-donor relationship, while arc failure occurs in the relationship between patient-donor pairs. Figure 4 illustrates the two types of failure. Node h failure means that there is a problem within patient-donor pair h, which means that any compatible arcs related to node h become incompatible. Arc (i,j) failure means that there is a problem between pairs i and j. Therefore, if arc (i,j) failure occurs, the compatibility between donor i and patient j becomes incompatible.

**Figure 5:**
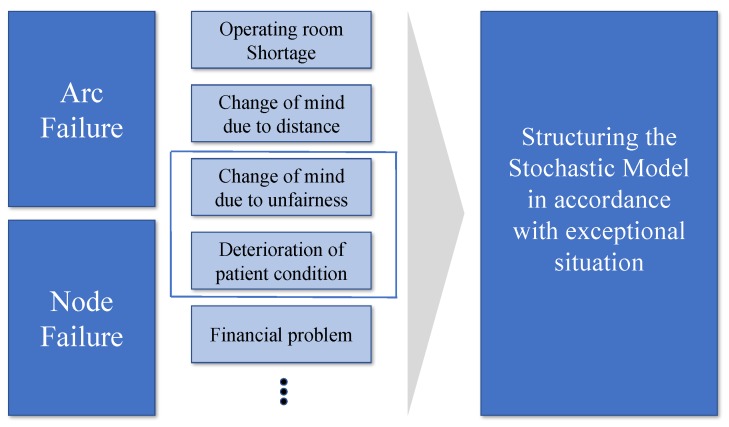
Various cases of KEP failures.

In this study, two representative examples of these failure types are presented: a change of mind due to perceived unfairness for arc failure and the deterioration of a patient’s condition for node failure. However, there are various other causes of failure that could also be considered, as shown in Figure 5.

A scenario k is a holistic and conceptual factor for possible cases of failure. It can be generated based on the classification that we propose and can be presented as below:

$$k\in K={\left\{\;k_{arc}^{ij},\;k_{node}^{ij}\right\}}_{\left(i,j\right)\in A}\;$$

where $k_{arc}^{ij}$ is a scenario of arc failure and $k_{arc}^{ij}$ is a scenario of node failure on arc (i,j)∈A. For notational simplicity, we simply denote $k_{arc}$ and $k_{node}$ respectively in case that it is evident to understand the arc (i,j) associated with a scenario. In a mathematical model, $p_{k},\;q_{ijk},$ and $y_{ijk}$ are realized based on the scenario k∈K, as mentioned in Section 2. The sum of the probabilities of these failures, that is $\underset{k\in K}{\sum }p_{k}$, should be 1. $q_{ijk}$ is a penalty value given to each node and arc depending on the scenario k. In this study, $q_{ijk_{node}}$ and $q_{ijk_{arc}}$ are proposed and calculated reflecting characteristics of their failure scenarios. $y_{ijk}$ is the second-stage decision variable depending on the scenario k.

● Node failure

The deterioration of the health of the patient in a patient-donor pair is selected as a representative example of node failure. If the health of the patient is so poor that it is dangerous to perform the operation, the solution set may be broken. Other possible causes of node failure include the patient’s death and financial problems. We introduce a penalty value based on the patient’s health condition. This value is only affected by the health status of the patient in individual pair. The penalty value is given as follows. 

$q_{ijk_{node}}:$ a penalty value of arc (i,j) depending on the health status of the patient in pair j regardless of the health status of the donor in pair i in scenario $k_{node}$ for ∀(i,j)∈A.

● Arc failure

A change of mind due to perceived unfairness is selected as a representative example of arc failure. If a pair feels that the exchange is unfair, they may change their mind and thus the solution set may be broken. In this paper, we have assumed that all patients and donors have certain health conditions. Therefore, based on the health status of the patient and donor, the level of compatibility was calculated. Other possible causes of arc failure are shortages of operating rooms and a change of mind due to long distance between two pairs. We introduce unfairness indicator $u_{ij}$ for arc (i,j)∈A  as follows:

$$\;u_{ij}=\;\frac{d_{j}\;}{w_{ij}\;}\;$$

where $d_{j}$ is the health status of the donor in pair j, $w_{ij}$ is the level of compatibility between pairs, and $u_{ij}$ is the value calculating unfair feelings between the relationship of pair i and pair j. From pair j’s perspective, it can be calculated by dividing the value that pair j receives into the value that pair j gives. Note that the larger the value of $d_{j}$ and the smaller the value of $w_{ij}$, the greater the value of $u_{ij}$. It is natural to feel an exchange is unfair if the value of donating a kidney is greater than the value of receiving one. We introduce a penalty value based on this perceived unfairness. The following definition and equation is used to determine the penalty value.

$q_{ijk_{arc}}$: a penalty value of arc (i,j) depending on the unfairness indicator $u_{ij}$ in scenario $k_{arc}$ for ∀(i,j)∈A.

$$\;\;\;\;\;q_{ijk_{arc}}\;=1-\;e^{\frac{u_{ij}}{c}},\forall \left(i,j\right)\in A\;$$

We remark that these parameters, $q_{ijk_{node}}$ and $q_{ijk_{arc}}$, have negative values imposed to penalize the effects of failure scenarios. When it comes to $q_{ijk_{node}}$, the worse the patient’s condition, the higher the penalty. For $q_{ijk_{arc}}$, as unfairness increases, the absolute value of this negative number increases exponentially. This means that the effect of penalty increases when an individual pair *j* feels more unfair. Note that $u_{ij}$ is divided by the constant c to scale the penalty value in the range of −1 and 0.

### 3.2. Model

In this section, we present as a base model the reduced edge assignment (EA) formulation first developed by Constantino et al. [15]. Our proposed stochastic programming model is then developed by adding new decision variables to this base model. These decision variables are related to the scenarios discussed earlier.

Let L be an upper bound on the possible number of cycles in graph G. The weight of edge $w_{ij}\;$ indicates the level of compatibility between the kidney from the donor in patient-donor pair i to the patient in pair j for (i,j)∈ A. Parameter c represents the maximum cycle length. The decision variables in the EA formulation are defined as follows:

$x_{ij}$: 1 if the patient of pair j receives a kidney from the donor of pair i, and 0 otherwise,

$z_{i}^{l}$: 1 if pair i is included in cycle l, and 0 otherwise.

With these decision variables, the reduced EA formulation can be presented as follows:

**Deterministic model (EA formulation):**

Maximize

(3a) $$\underset{\left(i,j\right)\in A}{\sum }w_{ij}x_{ij}\;$$

Subject to

(3b) $$\underset{j:\left(j,i\right)\in A}{\sum }x_{ji}=\;\underset{j:\left(i,j\right)\in A}{\sum }x_{ij}\;,\;\forall i\in V\;$$

(3c) $$\underset{j:\left(i,j\right)\in A}{\sum }x_{ij}\leq 1,\;\forall i\in V\;$$

(3d) $$\underset{i\in V}{\sum }z_{i}^{l}\leq c,\;\forall l\;\in 1,\ldots ,\;L\;$$

(3e) $$\underset{l\in 1,\ldots ,L}{\sum }z_{i}^{l}=\;\underset{j:\left(j,i\right)\in A}{\sum }x_{ij},\;\forall i\in V\;$$

(3f) $$z_{i}^{l}+x_{ij}\leq \;1+z_{j}^{l},\;\forall \left(i,j\right)\in A,\;l\in 1,\ldots ,L$$

(3g) $$z_{i}^{l}\leq z_{l}^{l},\forall i\in V,\;l\in 1,\ldots ,L,\;i>l\;$$

(3h) $$z_{i}^{l}=0,\;\forall i\in V,\;l\in 1,\ldots ,L,\;i\leq l\;$$

(3i) $$x_{ij}\in \left\{0,1\right\},\;\forall \left(i,j\right)\in A\;$$

(3j) $$z_{i}^{l}\in \left\{0,1\right\},\;\forall i\in V,\;l\in 1,\ldots ,L.$$

The objective function (3a) indicates that the total weights of the arcs involved in a kidney transplantation is maximized. This leads to a set of transplantations that maximizes the total compatibility of the given patient-donor pool to be selected by definition of weight $w_{ij}$. Note that if every $w_{ij}$ is set to 1, the model then maximizes the number of transplantations in the pool. Constraint (3b) states that the number of kidneys that the donor in pair i provides must be equal to the number of kidneys that the patient in pair i receives. Constraint (3c) ensures that the maximum possible number of kidneys that a donor can donate is 1. Constraint (3d) states that the length of a cycle in the pool must be less than or equal to c. Constraint (3e) assigns a transplantation to a cycle. Constraint (3f) states that, if the donor in pair i gives the patient in pair j a kidney and pair i is included in cycle l, then pair j must also be included in cycle l. Constraints (3g) and (3h) are additional constraints that eliminate duplicate solutions, as discussed in detail by Constantino et al. [15]. Constraints (3i) and (3j) set the decision variables $x_{ij}$ and $z_{j}^{l}$ as binary variables.

Based on the above formulation, we now develop a stochastic programming formulation for a KEP to consider failure within the KEP graph. As defined in Section 3.1, for each scenario k∈K, we let $p_{k}$ be the probability of the failure scenario occurrence, and $q_{ijk}$ be the penalty value given to each node and arc depending on the scenario k. We specify the additional decision variable $y_{ijk}$ for the two-stage stochastic programming model which is given as follows:

With the above decision variable, the two-stage stochastic programming model can be expressed as 

$y_{ijk}$: 1 if the patient of pair j receives a kidney from the donor of pair i in scenario k, and 0 otherwise.

(4) $$\begin{array}{cc} \mathrm{Maximize} & \underset{\left(i,j\right)\in A}{\sum }w_{ij}x_{ij}+E_{k\in K}\left(Q\left(y_{ijk},\;\xi_{k}\right)\right)\\ \mathrm{Subject}\;\mathrm{to} & \left(3b\right)-\left(3j\right), \end{array}$$

where $E_{k}$ is the average $Q\left(y_{ijk},\;\xi_{k}\right)$ over scenarios k∈K, and $Q\left(y_{ijk},\;\xi_{k}\right)$ is the optimal value of the following second-stage problem:

Maximize

(5a) $$\;\overset{}{\underset{k\in K}{\sum }}\underset{\left(i,j\right)\in A}{\sum }p_{k}q_{ijk}y_{ijk}\;$$

Subject to

(5b) $$x_{ij}\leq y_{ijk},\;\forall \left(i,j\right)\in A,\;k\in K$$

(5c) $$y_{ijk}\in \left\{0,1\right\},\;\forall \left(i,j\right)\in A,\;k\in K$$

The objective function (5a) in the second-stage problem returns the total penalty for the possible failure of a solution in scenario k. Constraint (5b) ensures that, in scenario k, the pairs which were matched in the first stage are considered. The common constraint (5c) sets the decision variable $y_{ijk}$ as a binary variable.

We remark that we can easily derive the deterministic equivalent version of the two-stage stochastic problem with discretized scenarios $k\;\in \;\left\{k_{node},\;k_{arc}\right\}$ where the corresponding probabilities are $p_{k}\in \left\{p_{k_{node}},p_{k_{arc}}\right\}$. (5a)–(5c) can be specified as below:

Maximize

(5a’) $$\;\underset{\left(i,j\right)\in A}{\sum }p_{k_{node}}q_{ijk_{node}}y_{ijk_{node}}+\underset{\left(i,j\right)\in A}{\sum }p_{k_{arc}}q_{ijk_{arc}}y_{ijk_{arc}}\;$$

Subject to

(5b’) $$\;x_{ij}\leq y_{ijk_{node}},\;\forall \left(i,j\right)\in A\;x_{ij}\leq y_{ijk_{arc}},\;\forall \left(i,j\right)\in A$$

(5c’) $$\;y_{ijk_{node}}\in \left\{0,1\right\},\;\forall \left(i,j\right)\in A\;y_{ijk_{arc}}\in \left\{0,1\right\},\;\forall \left(i,j\right)\in A$$

Finally, our proposed model, referred as a stochastic model, can be simply formulated as follows:

Stochastic model:

(6) $$\begin{array}{cc} \mathrm{Maximize} & \underset{\left(i,j\right)\in A}{\sum }w_{ij}x_{ij}+\underset{k\in K}{\sum }\underset{\left(i,j\right)\in A}{\sum }p_{k}q_{ijk}y_{ijk}\\ \mathrm{Subject}\;\mathrm{to} & \left(3b\right)-\left(3j\right)\\ & \left(5b\right),\;\left(5c\right) \end{array}$$

## 4. Computational Results

### 4.1. Experimental Design

In this section, we present the results of computational experiments on a KEP. The experiments were performed using an Intel^®^ Core™ i7-4650 CPU@1.70 GHz (Intel Corporation, Santa Clara, CA, USA), and Gurobi 6.5.2 (Gurobi Optimization, Beaverton, OR, USA) as an IP optimization solver. The experiments are designed to compare the proposed stochastic formulation to a previous deterministic model (i.e., the EA formulation).

We randomly generate ten data sets, with each set consisting of 50 incompatible pairs. Each pair has information about the blood type and the health status of the patient and donor. The ratio of each blood type was set at 0.3, 0.3, 0.3, and 0.1 for A, B, O, and AB, respectively. These probabilities were given based on the blood type distribution in South Korea [27]. In this paper, we have assumed that all patients and donors have certain health conditions and that, based on the health status of patient and donor, the level of compatibility was calculated. The health status of the patients and donors is classified as 1, 2, 3, or 4 with a probability of 0.25, with the higher the number, the better the health status. The level of compatibility is considered only when the ABO compatibility is satisfied, and is used to represent the weight $w_{ij}$. This has similar meaning to the probability of success, therefore this value is scaled in the range of [0, 1]. Table 1 shows the weight for two pairs (i,j) based on their health status.

Before the experiment, we assume that the probability of failure due to a change of mind would be greater than the probability of failure due to the condition deterioration and possible death of a patient and set the following scenario probabilities: $p_{k_{arc}}=0.8\;\mathrm{and}\;p_{k_{node}}=0.2$. We determined the penalty function in Section 2. For $q_{ijk}\;$ of node-failure scenario k, penalty values of 0, 0, −1, and −2 are assigned to P-group 1, P-group 2, P-group 3, and P-group 4, respectively. For $q_{ijk}$ of arc-failure scenario k, constant c was set at 15 to scale the value.

We compare two mathematical models in the computer simulations—a deterministic model as a benchmark vs. the proposed stochastic model. The aim is to determine whether the proposed model is able to reduce unfairness and whether it is robust when an exceptional event occurs.

### 4.2. Experimental Results and Managerial Insights

The objective function in the benchmark deterministic mathematical model is the sum of the weights, while the objective function in the stochastic mathematical model is the sum of the weights and the penalty function. In this experiment, we first compared the two models in terms of the sum of the weights and the sum of unfairness to determine whether the stochastic mathematical model preserves total utility (i.e., the sum of the weights) while reducing unfairness, and Table 2 shows the results. Secondly, we compared the two models in terms of robustness of solution when exceptional event occurs, and Table 3, Table 4, Table 5 and Table 6 show the results. Thirdly, we observed the distribution of unfairness indicator and the effect of unfairness threshold.

Table 2 presents the experimental results comparing total utility and total unfairness for the two models. W-GAP is the gap in total utility between the deterministic and stochastic models. U-GAP is the gap in total unfairness between the two models. The total utility of the proposed stochastic model is 4% lower on average than that of the deterministic model. This is because the stochastic model takes into account exceptional events and reflects the risk of uncertainty, thus there is a reduced number of connected patient-donor pairs. However, this decrease is insignificant, so it can be said that total utility has been preserved. The total unfairness of the stochastic model is 11.3% lower on average than that of the deterministic model. This indicates that the penalty function is effective at reducing the sense of unfairness in exchanges between patient-donor pairs. Kidney exchanges are sensitive to unfairness because they are part of a barter exchange program between patient-donor pairs. This result can be regarded as an effective improvement because it reduces the possibility of changes of mind by more fairly applying the principles of barter exchange while preserving total utility.

The stochastic optimization model is a scenario-based mathematical model. Experiments are thus conducted in which scenarios related to the patient’s death or the deterioration of the health of the patient are considered. If a patient cannot undergo surgery because of worsening health or death, the existing set of cycles is broken. If a cycle is broken, none of the operations involving the affected patient-donor pairs can be performed. This reduces the sum of the weights and increases the number of unconnected patient-donor pairs.

Table 3 presents the experimental results comparing the sum of the weights for the two models assuming that the patient’s health has deteriorated in the worst-case pair (i.e., Group 1). The deterministic model lost an average of 17.3% of its total utility (i.e., sum of the weights). However, the stochastic model only lost an average of 4.2%. This indicates that, when node failure occurs, the stochastic model minimizes the damage when compared to the deterministic model.

Table 4 displays the number of pairs that belong to the solution set obtained from the mathematical model for 50 patient-donor pairs and the number of broken pairs before and after node failure. Before failure, the deterministic model connects 2.2 more pairs (4.4%) on average than does the stochastic model; however, after failure, the stochastic model connects an average of 4 more pairs (8%) than the deterministic model. The broken pairs column represents the gap of broken pairs before and after node failure for the two models. The stochastic models lose an average of 1.8 pairs (3.6%) after failure, while the deterministic models lose an average of 8 pairs (16%). The results show that the stochastic model is more robust in terms of preserving the solutions in the face of failure than the deterministic model.

Table 5 compares the sum of the weights for the two models for cases where there is a change of mind due to perceived unfairness between pair (i, j)∈A whose $u_{ij}$ is more than the threshold of 5.5. The deterministic model loses an average of 34.1% of its total utility, while the stochastic model loses an average of 6.7%. This indicates that, when arc failure (e.g., a change of mind due to perceived unfairness) occurs, the stochastic model is more robust to failure than the deterministic model.

Table 6 presents the number of connected pairs before and after arc failure. Similar to the case of node failure, the stochastic model connects more pairs (8, representing 16% of the total number of pairs) on average after arc failure than the deterministic model, even though it connects slightly fewer before failure. The stochastic model loses an average of 2.8 pairs (5.6%) after failure, while the deterministic model loses 12.6 pairs (25.2%). This indicates that the stochastic model preserves the solutions better than the deterministic model after arc failure. 

**Figure 6:**
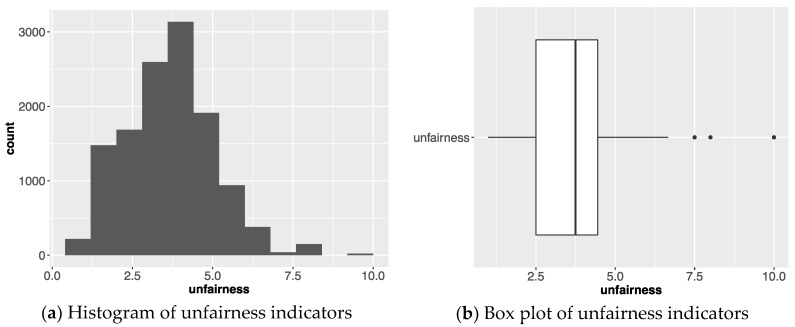
The distribution of unfairness indicators.

Figure 6 presents the distribution of an unfairness indicator. By the definition of $u_{ij}$ in Section 3.1, $u_{ij}$ has a value in the range of 1 and 12 because $w_{ij}$ has a value in the range of 0.3 and 1, and $d_{j}$ has value in the range of 1 and 4. Figure 6a is the histogram of an unfairness indicator and Figure 6b is the box plot of unfairness. These two graphs show that most of the unfairness indicator values are in the range of 2 and 8. We conducted the next experiment based on this observation.

**Figure 7:**
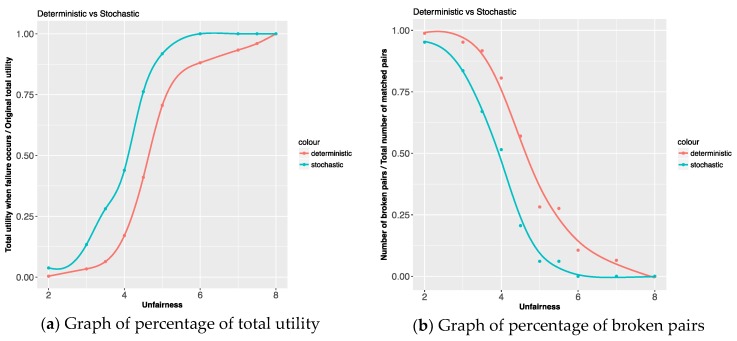
The effect of the unfairness threshold.

We now discuss the effect of unfairness threshold on deterministic and stochastic models. We assumed that a change of mind (arc failure) occurs if the unfairness indicator has a higher value than the unfairness threshold. In the perspective of the entire model, the larger the value of the unfairness threshold, the more lenient the model is (i.e., the lower it is, the stricter the model is). We conducted the experiment showing the effect of the unfairness threshold on the deterministic and stochastic model by changing the threshold value from 2 to 8. Figure 7a graph has an x-axis of unfairness and a y-axis of percentage value, which is total utility when failure occurs divided by original total utility. In stochastic model, when the threshold value is above 6, the percentage value is 1. This means that there are no two pairs having an unfairness indicator value higher than 6. On the other hand, in deterministic model, when the threshold value is above 6, the percentage value is near 0.88. This means that there are several pairs having an unfairness indicator value higher than 6. These results show that the performance of our proposed stochastic model is better in the sense that it maintains pairs with appropriate values of the unfairness indicator. In both deterministic and stochastic models, when the threshold value is under 5, the percentage value drops very fast, implying that there are many pairs having an unfairness indicator value lower than 5. This observation can be confirmed by Figure 6. Figure 7b has an x-axis of unfairness and a y-axis of the number of broken pairs divided by the total number of matched pairs when failure occurs. Figure 7b shows the corresponding results in Figure 7a. The stochastic model is more effective than the deterministic model in that it selects and maintains pairs with an appropriate range of the unfairness indicator.

## 5. Conclusions

In this paper, stochastic programming is proposed as a method for incorporating uncertainty into KEP models. Based on the compact EA formulation, we develop a two-stage SP model by adding second-stage decision variables and parameters. In order to consider exceptional events in the model, we classify specific failure scenarios based on two types of failure: node failure and arc failure. The deterioration of the patient’s condition and a change of mind due to perceived unfairness are chosen as representative examples of node and arc failure, respectively. While previous studies have defined fairness as social fairness, we define it as personal fairness and consider how satisfied individual pairs feel. An indicator of unfairness is introduced; as this number increases, the penalty value imposed in the second stage increases. Computational simulations are run to compare an existing deterministic model with our proposed stochastic model. From these, it is shown that the stochastic model reduces overall perceived unfairness while maintaining total utility. We also demonstrate that, when the two failure scenarios occur, the stochastic model is more robust in terms of preserving the solutions. We believe that the proposed model could help to be utilized in the integrated KEP IT healthcare platform in South Korea to obtain optimized fair KEP exchange plans.

In future research, the two-stage SP model proposed in this paper can be tested with other types of failure, thus leading to a more robust model. Bertsimas et al. [28] suggested the widely used Robust Optimization (RO) model, which made it possible to generate robust counterpart of Linear Programming (LP) problems. Büsing et al. [29] also suggested robust counterpart of LP with uncertain coefficient matrix. These two approaches provide insight for future work because SP and RO have overlap in that they can be linearized. Robust models for KEPs have been presented in various perspectives by Dickerson [30]. D’Andreagiovanni et al. [31] considered data uncertainty in topology and applied RO. They applied RO to the Network Problem, in which our work belongs to. RO in KEP or Network Problem would be the topic for the future work.

Because a KEP is related to surgery, which is associated with significant time and money costs, robustness to failure is an important consideration. Our study only considers a static pool, but other studies have analyzed pools that move dynamically over time [32,33]. Therefore, even more promising results may be achieved if we incorporate patient-donor pairs that flow in time.

## Figures and Tables

**Table 1:** The level of compatibility based on health status.

Patient *j*/Donor *i*	D-Group 1	D-Group 2	D-Group 3	D-Group 4
P-group 1	0.30	0.40	0.50	0.70
P-group 2	0.40	0.60	0.70	0.80
P-group 3	0.50	0.70	0.85	0.90
P-group 4	0.70	0.80	0.90	1.00

**Table 2:** Comparison of the two models in terms of total weight and total unfairness.

Dataset	Sum of Weights			Sum of Unfairness		
	Deterministic	Stochastic	W-GAP	Deterministic	Stochastic	U-GAP
Dataset_1	37.6	36.3	3.5%	186.6	171.1	8.3%
Dataset_2	35.9	35.3	1.7%	180.4	170.8	5.3%
Dataset_3	35.9	32.9	8.4%	178.4	148.4	16.8%
Dataset_4	33.1	32.1	3.0%	163.5	148.4	9.2%
Dataset_5	27.7	26.5	4.3%	145.5	129.1	11.3%
Dataset_6	34.6	33.9	2.0%	173.0	159.5	7.8%
Dataset_7	32.2	31.4	2.5%	152.5	133.1	12.7%
Dataset_8	34.0	32.5	4.4%	174.9	143.7	17.8%
Dataset_9	34.5	32.4	6.1%	180.3	153.4	14.9%
Dataset_10	29.4	28.3	3.7%	135.2	123.4	8.7%
Average	33.5	32.2	4.0%	167.0	148.1	11.3%

**Table 3:** Comparison of the sum of weights when there is a deterioration in the patient’s health.

Dataset	Sum of Weights					
	Deterministic Model			Stochastic Model		
	Before Failure	After Failure	W-GAP	Before Failure	After Failure	W-GAP
Dataset_1	37.6	33	12.2%	36.3	36.3	0.0%
Dataset_2	35.9	31.6	12.0%	35.3	33.8	4.2%
Dataset_3	35.9	26.4	26.5%	32.9	32.9	0.0%
Dataset_4	33.1	23.6	28.7%	32.1	26.9	16.2%
Dataset_5	27.7	21.7	21.7%	26.5	21.9	17.4%
Dataset_6	34.6	30.4	12.1%	33.9	33.9	0.0%
Dataset_7	32.2	31.1	3.4%	31.4	31.4	0.0%
Dataset_8	34	25.8	24.1%	32.5	30.9	4.9%
Dataset_9	34.5	26.9	22.0%	32.4	32.4	0.0%
Dataset_10	29.4	26.1	11.2%	28.3	28.3	0.0%
Average	33.5	27.7	17.4%	32.2	30.9	4.0%

**Table 4:** Comparison of the number of matched pairs when the patient’s health deteriorates.

Dataset	Total Number of Matched Pairs				Broken Pairs	
	Before Failure		After Failure		Gap (Before-After)	
	Deterministic	Stochastic	Deterministic	Stochastic	Deterministic	Stochastic
Dataset_1	48	46	42	46	6	0
Dataset_2	47	46	41	44	6	2
Dataset_3	50	45	37	45	13	0
Dataset_4	44	42	33	34	11	8
Dataset_5	36	34	28	28	8	6
Dataset_6	46	45	40	45	6	0
Dataset_7	45	44	43	44	2	0
Dataset_8	45	43	34	41	11	2
Dataset_9	49	45	37	45	12	0
Dataset_10	40	38	35	38	5	0
Average	45.0	42.8	37.0	41.0	8.0	1.8
Percentage of matched pairs ^1^	90.0%	85.6%	74.0%	82.0%	16.0%	3.6%

^1^ Number of matched pairs divided by the total number of pairs when (node or arc) failures occurs.

**Table 5:** Comparison of the sum of the weights when a pair changes its mind.

Dataset	Total Number of Unmatched Pairs					
	Deterministic Model			Stochastic Model		
	Before Failure	After Failure	W-GAP	Before Failure	After Failure	W-GAP
Dataset_1	37.6	31.5	12.2%	36.3	36.3	0.0%
Dataset_2	35.9	30.1	12.0%	35.3	33.7	4.2%
Dataset_3	35.9	26.6	26.5%	32.9	30.7	6.7%
Dataset_4	33.1	21.4	28.7%	32.1	27.4	14.6%
Dataset_5	27.7	19.2	21.7%	26.5	24.1	9.1%
Dataset_6	34.6	23.2	30.4%	33.9	31.5	7.1%
Dataset_7	32.2	24.4	31.1%	31.4	29.2	7.0%
Dataset_8	34.0	18.0	25.8%	32.5	30.9	4.9%
Dataset_9	34.5	17.2	26.9%	32.4	30.0	7.4%
Dataset_10	29.4	25.0	26.1%	28.3	26.1	7.8%
Average	33.5	22.1	34.1%	32.2	30.0	6.7%

**Table 6:** Comparison of the number of unmatched pairs when a pair changes their mind.

Dataset	Total Number of Matched Pairs				Broken Pairs	
	Before Failure		After Failure		Gap (Before-After)	
	Deterministic	Stochastic	Deterministic	Stochastic	Deterministic	Stochastic
Dataset_1	48	46	42	46	6	0
Dataset_2	47	46	41	44	6	2
Dataset_3	50	45	37	45	13	0
Dataset_4	44	42	33	34	11	8
Dataset_5	36	34	28	28	8	6
Dataset_6	46	45	40	45	6	0
Dataset_7	45	44	43	44	2	0
Dataset_8	45	43	34	41	11	2
Dataset_9	49	45	37	45	12	0
Dataset_10	40	38	35	38	5	0
Average	45.0	42.8	37.0	41.0	8.0	1.8
Percentage of matched pairs	90.0%	85.6%	74.0%	82.0%	16.0%	3.6%
